# Rare Gain-of-Function *KCND3* Variant Associated with Cerebellar Ataxia, Parkinsonism, Cognitive Dysfunction, and Brain Iron Accumulation

**DOI:** 10.3390/ijms22158247

**Published:** 2021-07-31

**Authors:** Cheng-Tsung Hsiao, Thomas F. Tropea, Ssu-Ju Fu, Tanya M. Bardakjian, Pedro Gonzalez-Alegre, Bing-Wen Soong, Chih-Yung Tang, Chung-Jiuan Jeng

**Affiliations:** 1 Department of Neurology, Taipei Veterans General Hospital, Taipei 11217, Taiwan; hsiaoct26@gmail.com; 2 Department of Physiology, College of Medicine, National Taiwan University, Taipei 10051, Taiwan; d01441001@ntu.edu.tw; 3 Department of Neurology, School of Medicine, National Yang Ming Chiao Tung University, Taipei 11221, Taiwan; bwsoong@gmail.com; 4 Brain Research Center, National Yang Ming Chiao Tung University, Taipei 11221, Taiwan; 5 Department of Neurology, Perelman School of Medicine, University of Pennsylvania, Philadelphia, PA 19104, USA; thomas.tropea@pennmedicine.upenn.edu (T.F.T.); Tanya.Bardakjian@pennmedicine.upenn.edu (T.M.B.); Pedro.Gonzalez-Alegre@pennmedicine.upenn.edu (P.G.-A.); 6 Institute of Anatomy and Cell Biology, College of Medicine, National Yang Ming Chiao Tung University, Taipei 11221, Taiwan; 7 Department of Neurology, Shuang Ho Hospital and Taipei Neuroscience Institute, Taipei Medical University, Taipei 11031, Taiwan

**Keywords:** spinocerebellar ataxia, parkinsonism, molecular genetics, channelopathy, iron homeostasis

## Abstract

Loss-of-function mutations in the K_V_4.3 channel-encoding *KCND3* gene are linked to neurodegenerative cerebellar ataxia. Patients suffering from neurodegeneration associated with iron deposition may also present with cerebellar ataxia. The mechanism underlying brain iron accumulation remains unclear. Here, we aim to ascertain the potential pathogenic role of *KCND3* variant in iron accumulation-related cerebellar ataxia. We presented a patient with slowly progressive cerebellar ataxia, parkinsonism, cognitive impairment, and iron accumulation in the basal ganglia and the cerebellum. Whole exome sequencing analyses identified in the patient a heterozygous *KCND3* c.1256G>A (p.R419H) variant predicted to be disease-causing by multiple bioinformatic analyses. In vitro biochemical and immunofluorescence examinations revealed that, compared to the human K_V_4.3 wild-type channel, the p.R419H variant exhibited normal protein abundance and subcellular localization pattern. Electrophysiological investigation, however, demonstrated that the K_V_4.3 p.R419H variant was associated with a dominant increase in potassium current amplitudes, as well as notable changes in voltage-dependent gating properties leading to enhanced potassium window current. These observations indicate that, in direct contrast with the loss-of-function *KCND3* mutations previously reported in cerebellar ataxia patients, we identified a rare gain-of-function *KCND3* variant that may expand the clinical and molecular spectra of neurodegenerative cerebellar disorders associated with brain iron accumulation.

## 1. Introduction

In neurons, the voltage-gated potassium (K^+^) channel subunit K_V_4.3 is localized in the somatodendritic compartment and contributes to the generation of A-type K^+^ currents essential for regulating neuronal excitability and action potential firing [1,2]. The K_V_4.3 channel is also significantly expressed in the heart, where it plays a crucial role in mediating the transient outward K^+^ current that shapes the early repolarization phase of the cardiac action potential [3,4,5]. Perturbation of K_V_4.3 channel properties may therefore substantially affect neuronal and/or cardiac functions.

Loss-of-function mutations in the human *KCND3* gene, which encodes the K_V_4.3 channel, have been associated with spinocerebellar ataxia type 19 and 22 (SCA19/22), a clinically heterogeneous group of neurodegenerative disorders characterized by variable degrees of cerebellar ataxia, parkinsonism, cognitive dysfunction, epilepsy, and extrapyramidal signs (MIM#607346) [6,7,8,9,10]. In contrast, gain-of-function mutations in the *KCND3* gene are linked to cardiac arrhythmia, the Brugada syndrome [11]. Nonetheless, some of the cardiopathogenic gain-of-function *KCND3* mutations may also be related to neurological disorders such as ataxia, cognitive dysfunction, and epilepsy [10,12,13,14].

In the brain, iron homeostasis is crucial for maintaining key physiological functions such as the synthesis of myelin and neurotransmitters [15]. In healthy aging and neurodegenerative diseases, excessive concentration of free iron leads to iron accumulation in brain regions including the basal ganglia and the cerebellum [15,16,17]. Patients suffering from neurodegeneration associated with iron deposition, which can be detected by magnetic resonance imaging (MRI), may develop movement disorders, cognitive decline, and cerebellar ataxia [15,18].

The detailed mechanisms underlying brain iron deposition remain unresolved, as only two of the 15 known causal genes for neurodegeneration with brain iron accumulation are directly linked to iron homeostasis (MIM#117700 and MIM#606159) [15,19]. Intriguingly, excessive iron accumulation has also been observed in patients suffering from neurodegenerative cerebellar ataxia disorders [20,21,22]. It remains unclear, however, whether ataxia-related *KCND3* variants may be associated with brain iron accumulation. In this study, we report the identification of a rare *KCND3* c.1256G>A (p.R419H) variant in a patient with cerebellar ataxia, parkinsonism, cognitive impairment, and brain iron accumulation. Further biochemical and electrophysiological analyses suggest that the *KCND3* variant leads to a gain-of-function K_V_4.3 channel phenotype. Our findings expand the clinical and molecular spectra of neurodegenerative cerebellar ataxia associated with iron deposition in the brain.

## 2. Results

### 2.1. Case Presentation

**Figure 1:**
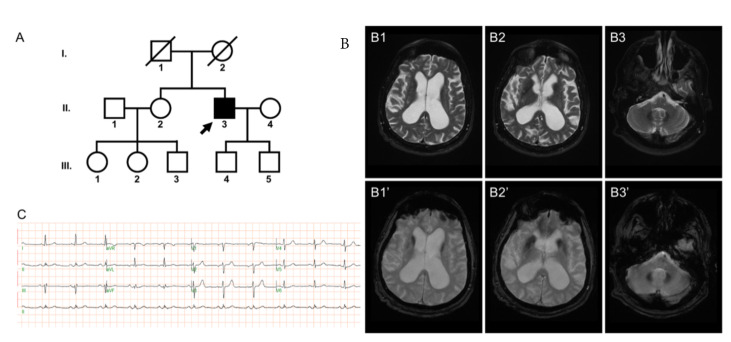
Clinical features of the patient with the *KCND3* c.1256G>A (p.R419H) variant. (**A**) Pedigree of the patient characterized in current study. In this pedigree, the heterozygous *KCND3* variant was detected in the male individual II.3, who is now 70 years old. The patient’s father (I.1) passed away in his seventies due to heart failure, and his mother (I.2) in her eighties due to chronic obstructive pulmonary disease. He has a sister (II.2) who is five years older than him. His two sons (III.4 and III.5) are now in their thirties. Arrow denotes the index case who harbors the *KCND3* c.1256G>A (p.R419H) variant. Filled symbol represents symptomatic member. Open symbols indicate unaffected individuals. Circles stand for females. Squares correspond to males. Diagonal lines refer to the deceased. (**B**) Neuroimages of the patient. The axial T2-weighted (**B1**–**B3**) and the corresponding gradient-echo sequence (**B1′**–**B3′**) images demonstrate hypointensity in bilateral caudate nuclei (**B1**,**B1′**) and lentiform nuclei (**B2**,**B2′**) of the basal ganglia, as well as in bilateral dentate nuclei of the cerebellum (**B3**,**B3′**). (**C**) Standard 12-lead ECG indicates normal sinus rhythm.

The proband is a male with a past medical history of anxiety disorder who first presented to the University of Pennsylvania (UPenn) Movement Disorders Clinic at the age of 69 years with a chief complaint of gait dysfunction. He reported normal development with the exception of mild gait instability with occasional falls while playing sports as a child and throughout adulthood. In his mid-60s, the patient’s balance deteriorated with an increased frequency of falls, and he developed urinary urgency with incontinence, and cognitive changes characterized by poor recall, naming, personality changes, disinhibition, and inappropriate joking. The patient was born in a non-consanguineous pedigree with no reported family history of neurological diseases (Figure 1A). His parents are deceased due to non-neurological causes. No significant neurological deficit was noted for the patient’s sibling and offspring.

The neurological examination revealed cerebellar ataxia and mild parkinsonism, characterized by masked facial expression, perioral dyskinesias, mild intention tremor and mild dysmetria, bilateral limb ataxia with dysdiadochokinesia, lower greater than upper extremity bradykinesia, paratonia, upright rigid posture, wide-based gait with short stride length, and mild tandem gait impairment (Supplementary Video S1; Supplementary Figure S1). He achieved 25/30 points on the Montreal Cognitive Assessment. Neuropsychological evaluation at the age of 69 demonstrated average overall intellectual functioning and verbal skills, with high average perceptual reasoning abilities. Executive dysfunction was also evident with notable impairments in processing speed, complex sequencing, inhibition, novel problem-solving, and conceptual reasoning and perseveration, as well as set loss errors, impulsivity, confabulation, verbosity, tangentiality, conflation, poor organization/planning, and variable self-monitoring. Memory retention was intact.

Prior to presenting to the clinic, a presumptive diagnosis of normal pressure hydrocephalus prompted high-volume cerebrospinal fluid drainage (25 cc, opening pressure 15 cm-H_2_O) without improvement in gait or memory problems. Serological evaluation for ataxia was unrevealing. MRI of the brain on a 1.5 T scanner demonstrated two main features: (i) diffuse cerebral atrophy with ventriculomegaly and mild white matter disease; (ii) iron deposition in pallidal, caudate, and dentate nuclei (Figure 1B). A standard electrocardiogram (ECG) examination showed normal sinus rhythm (Figure 1C).

Gene panel screening revealed no detectable relevant sequence variant in 17 genes known to be linked to neurodegeneration with brain iron accumulation (*ATP13A2*, *C19orf12*, *COASY*, *CP*, *DCAF17*, *FA2H*, *FTL*, *FUCA1*, *KIF1A*, *PANK2*, *PLA2G6*, *SCP2*, *SLC39A14*, *SQSTM1*, *TRIM32*, *VPS13A*, *EDR45*). Subsequent clinical exome sequencing plus mitochondrial sequencing led to the identification of a heterozygous variant in exon 3 of *KCND3* (c.1256G>A; p.R419H) (NM_004980.4).

### 2.2. In Silico Pathogenicity

We employed population databases and bioinformatics analyses to evaluate the pathogenicity of the identified *KCND3* variant (Table 1). In both the total and non-Finnish European population in the genome Aggregation Database (gnomAD), the estimated allele frequency of the c.1256G>A (p.R419H) variant is less than 0.0001, suggesting that this is a rare *KCND3* variant.

The c.1256G>A (p.R419H) variant was predicted to be damaging/disease-causing based on several in silico prediction tools. The CADD program [23] estimated a Phred score of 28.2, suggesting the variant may be more deleterious than 99.85% of the other variants in the genome. Polyphen-2 predicted a probability score as high as 1, implying that the variant is probably damaging [24]. The programs SNPs&GO [25] and SIFT [26] predicted the variant is disease-related and deleterious, respectively. Moreover, MutationTaster [27] also revealed a high probability score close to 1, consistent with the idea that the rare *KCND3* c.1256G>A (p.R419H) variant may be disease-associated.

### 2.3. Lack of Effect of the p.R419H Variant on K_V_4.3 Protein Expression and Localization

**Figure 2:**
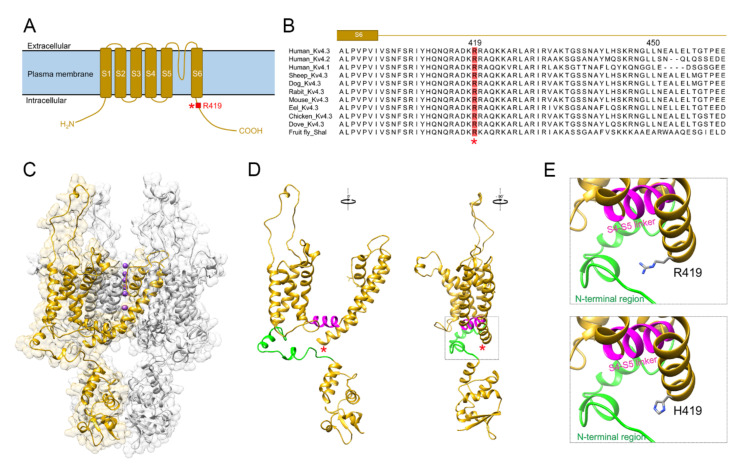
Protein structure modeling of human K_V_4.3 WT and the p.R419H variant. (**A**) Schematic representation of the membrane topology of a single K_V_4.3 subunit, highlighting the cytoplasmic localization of the residue p.R419 in the proximal carboxyl-terminal region close to the S6 transmembrane segment. (**B**) Amino acid sequence alignment of human K_V_4.3 with various K_V_4-related proteins from multiple animal species. The highly conserved p.R419 residue is decorated in red and marked with an asterisk. (**C**–**E**) Homology model of K_V_4.3 was based on the crystal structure of the voltage-dependent K^+^ channel K_V_1.2 (PBD ID: 3LUT) and generated using UCSF Chimera interfaced with Modeller. (**C**) Tetrameric structure of the K_V_4.3 channel. One K_V_4.3 monomer is displayed as ribbon in gold, with the other subunits in gray. The shadow refers to the surface representation of the homology model. Purple spheres denote K^+^ passing through the pore region. (**D**) (*Left panel*) The single K_V_4.3 subunit in gold in (**C**) is shown here. (*Right panel*) The same K_V_4.3 subunit is viewed with 90-degree rotation clockwise. The red asterisk highlights the location of the residue p.R419. The S4-S5 linker is shown as magenta helix, and a portion of the amino (N)-terminal region is colored in green. The area enclosed by dotted lines in the right panel is enlarged for inspection in (**E**). (**E**) Comparison of the side-chain structure of the p.R419 WT and the p.H419 variant with respect to the local microenvironment.

We went on to investigate the potential effect of the p.R419H variant on the in vitro property of human K_V_4.3 channel. The K_V_4.3 subunit comprises an intracellular amino-terminal domain, six transmembrane segments (S1–S6) containing a K^+^-conducting pore loop in the S5–S6 linker region, and a cytoplasmic carboxyl-terminal domain (Figure 2A). The p.R419 residue is localized in the intracellular carboxyl-terminal region, close to the S6 transmembrane segment. p.R419 is a highly conserved residue across various K_V_4 channel protein orthologs from multiple animal species (Figure 2B), implying an evolutionary importance in the structure and function of K_V_4.3 channel. Substitution of the positively charged, aliphatic amino acid arginine at residue 419 with the imidazole-containing histidine results in a sizable reduction in the mean side-chain volume by about 17.3% (from 202 Å^3^ to 167 Å^3^) (Figure 2C–E).

**Figure 3:**
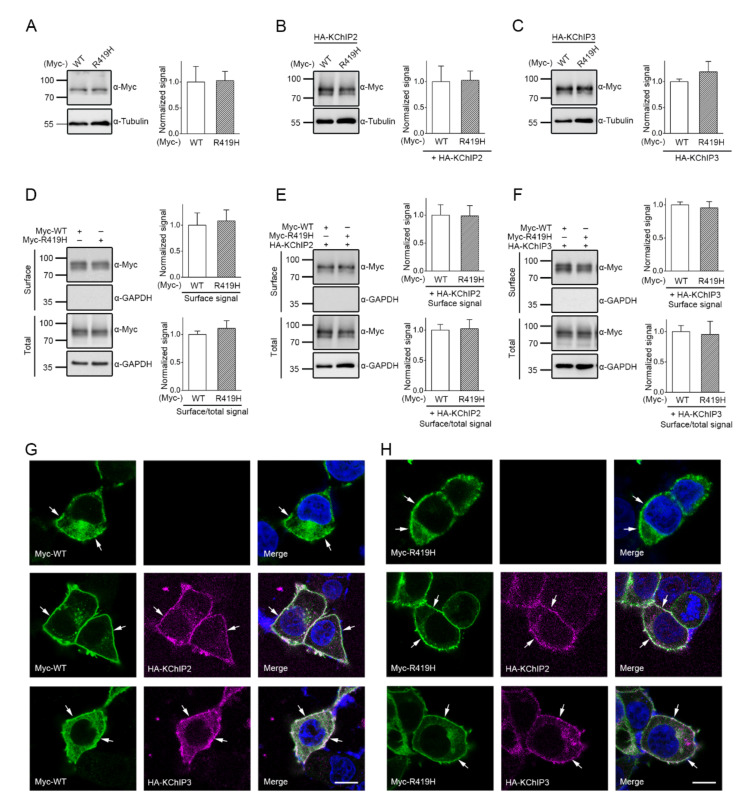
Biochemical and immunofluorescence characterizations of human K_V_4.3 WT and the p.R419H variant. Myc-K_V_4.3 subunits were expressed in HEK293T cells in the absence or presence of auxiliary HA-KChIP2 and -KChIP3 subunits. (**A**–**C**) (*Left panels*) Representative immunoblots. Proteins were detected with anti-Myc (α-Myc) and anti-tubulin (α-Tubulin) antibodies. Molecular weight markers (in kDa) are labeled to the left. (*Right panels*) Quantitative analyses indicating comparable protein level of the WT and the p.R419H K_V_4.3. Total K_V_4.3 protein density was standardized with cognate tubulin level, followed by normalization with respect to the WT (*n* = 4–6). (**D**–**F**) Surface biotinylation analyses for Myc-K_V_4.3 subunits in the absence (**D**) or presence of auxiliary HA-KChIP2 (**E**) and -KChIP3 (**F**) subunits. Representative immunoblots and quantitative analyses for the surface signals are shown at the left and right panels, respectively (*n* = 4). The surface/total ratio corresponds to membrane trafficking efficiency. (**G**,**H**) Representative immunofluorescence images demonstrating similar subcellular localization pattern of K_V_4.3 WT and p.R419H (*green*) in the absence (*top panels*) or presence of KChIP2 (*middle panels*) and KChIP3 (*bottom panels*) (*magenta*). Cell nuclei were counterstained with DAPI (*blue*). Arrows denote membrane localization. Merged images are shown in the right panels. Scale bar: 10 μm.

To assess the pathophysiological significance of the p.R419H variant, we began by determining whether the p.R419H affects protein homeostasis of K_V_4.3 channels. K_V_4.3 wild-type (WT) and p.R419H proteins were individually expressed in HEK293T cells. As shown by the immunoblots depicted in Figure 3A–C, regardless of the absence or presence of the auxiliary K^+^ channel interacting protein 2 and 3 (KChIP2/KChIP3) subunits, no significant difference in the total protein level was observed between K_V_4.3 WT and the p.R419H variant. Moreover, surface biotinylation analyses demonstrated that K_V_4.3 WT and the p.R419H variant displayed comparable protein abundance at the plasma membrane in the absence/presence of the auxiliary KChIP2/KChIP3 subunits (Figure 3D–F). Consistent with these biochemical observations, immunofluorescence analyses also indicated that, whether the auxiliary KChIP2/KChIP3 subunits were present or not, the majority of K_V_4.3 WT and the p.R419H variant exhibited similar punctate staining pattern at the cell surface, consistent with the presence of effective plasma membrane-localization for both proteins (Figure 3G,H). Together, these results suggest that the p.R419H variant does not appear to detectably affect protein expression and subcellular localization of K_V_4.3 channels.

### 2.4. Dominant Gain-of-Function Effect of the p.R419H Variant on K_V_4.3 Channel Function

**Figure 4:**
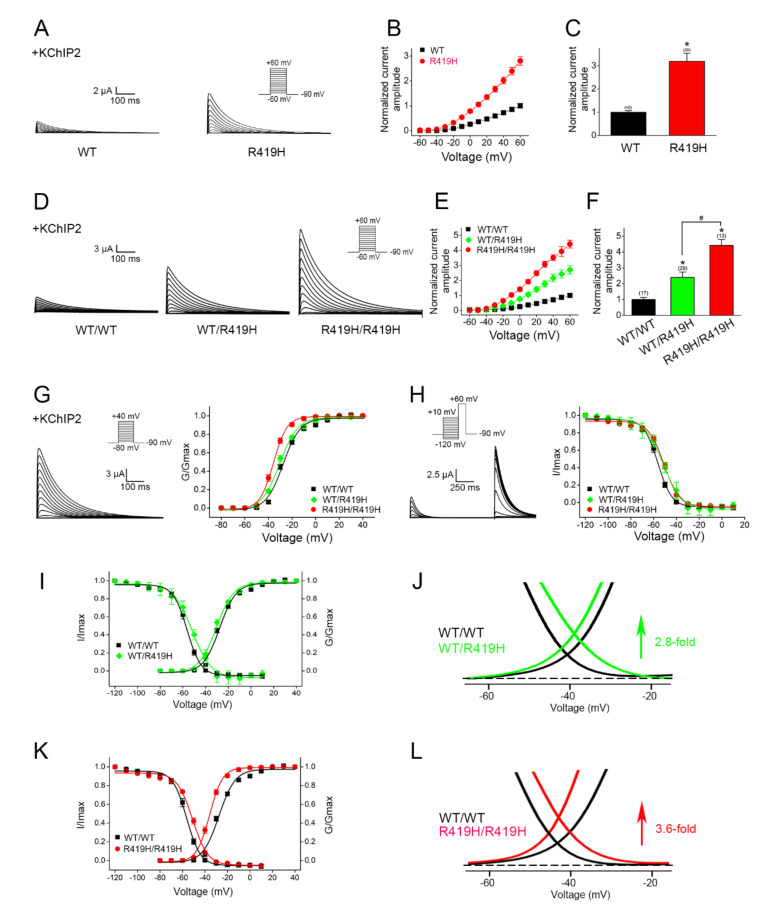
Characterization of the ion channel function of human K_V_4.3 WT and the p.R419H variant. K_V_4.3 was co-expressed with the auxiliary KChIP2 subunit in the 1:1 molar ratio in *Xenopus* oocytes. (**A**–**C**) Compared to K_V_4.3 WT, the p.R419H variant is associated with a significantly higher functional expression level. (**A**) Representative K^+^ current traces recorded from homotetrameric K_V_4.3 WT and p.R419H channels. Current traces were induced by a voltage protocol comprising test potentials ranging from –60 mV to +60 mV in 10-mV steps. (**B**) Normalized peak current amplitudes were plotted against matching test pulse potentials. For each current trace induced by a test pulse potential, the peak current amplitude was measured, followed by normalization with respect to the corresponding mean peak current amplitude at +60 mV of K_V_4.3 WT. (**C**) Statistical comparison of the normalized peak current amplitude at +60 mV. Mean normalized peak current amplitude at +60 mV: WT, 1 ± 0.1; p.R419H, 3.2 ± 0.3. Asterisks denote significant difference from the WT control (*p* < 0.05). Numbers in parentheses refer to the amount of cells analyzed for each K_V_4.3 construct. (**D**–**F**) The p.R419H variant exerts a dominant effect on the functional expression of K_V_4.3 channels. (**D**) Representative current traces recorded from K_V_4.3 WT/WT homotetramers, WT/R419H heterotetramers, and R419H/R419H homotetramers. (**E**) Normalized peak current-voltage plot. (**F**) Statistical comparison. Mean normalized peak current amplitude at +60 mV: WT/WT, 1 ± 0.1; WT/R419H, 2.4 ± 0.3; R419H/R419H, 4.4 ± 0.4. Asterisks denote significant difference from the WT/WT control (*p* < 0.05). The pound sign (#) indicates significant difference from the WT/R419H heterotetramer (*p* < 0.05). Numbers in parentheses refer to the amount of cells analyzed for each K_V_4.3 expression condition. (**G**,**H**) The p.R419H variant confers a substantial alteration in the voltage-dependent gating property of K_V_4.3 channels. (*Left panels*) Representative K^+^ current traces in response to the test pulse protocol for assessing voltage-dependent activation (**G**) and inactivation (**H**) of K_V_4.3 channels. (*Right panels*) Comparison of the steady-state voltage-dependent activation (**G**) and inactivation (**H**) curves of K_V_4.3 WT/WT homotetramers, WT/R419H heterotetramers, and R419H/R419H homotetramers. See Table 2 for detailed voltage-dependent gating parameters. (**I**–**L**) The p.R419H variant confers a prominent increase in K_V_4.3 window current. (**I**,**K**) Combination of the steady-state activation (**G**) and inactivation (**H**) curves of the indicated K_V_4.3 homotetramers and heterotetramers. (**J**,**L**) The triangular area underneath the overlap of corresponding activation and inactivation curves defines the size of the window current. Both the WT/R419H heterotetramer and the R419H/R419H homotetramer are associated with a significant increase in the window current size of K_V_4.3 channels.

Next, we examined the impact of the p.R419H variant on K_V_4.3 channel function by performing electrophysiological analyses. Surprisingly, compared to its WT counterpart, K_V_4.3 channels harboring the p.R149H variant were associated with a more than three-fold increase in the K^+^ current level (Figure 4A–C), with no apparent change in the channel activation and inactivation kinetics. This result implies that the p.R419H variant may substantially promote K_V_4.3 channel function.

A functional voltage-gated K^+^ channel is formed by the assembly of four K^+^ channel protein subunits (tetramer). Given the fact that the proband carries the heterozygous c.1256G>A (p.R419H) variant in the *KCND3* gene and that K_V_4.3 WT and the p.R419H variant appear to display comparable protein expression levels (Figure 3), it is likely that K_V_4.3 WT and p.R419H variant subunits may co-assemble and form heterotetrameric K_V_4.3 channels in native cells in the patient. We therefore asked whether the p.R419H variant may exert a dominant effect on the functional expression of its K_V_4.3 WT counterpart. To address this important issue, we co-expressed K_V_4.3 WT and WT (WT/WT homotetramer), WT and p.R419H (WT/R419H heterotetramer), or p.R419H and p.R419H (R419H/R419H homotetramer) in the same cell, followed by comparing their functional properties. As outlined in Figure 4D–F, the mean current amplitude of K_V_4.3 WT/R419H heterotetramers is more than two-fold lager than that of WT/WT homotetramers; moreover, the K^+^ current level of R419H/R419H homotetramers is substantially higher than that of WT/R419H heterotetramers. This R419H-dependent, progressive increase in K^+^ current level strongly argues that, in WT/R419H heterotetramers, the p.R419H variant is associated with a dominant effect on the functional expression of K_V_4.3 channels.

To further explore the potential mechanism underlying the observed enhanced current amplitude associated with the p.R419H variant, we analyzed the voltage-dependent gaiting property of K_V_4.3 channels comprising WT/WT homotetramers, WT/R419H heterotetramers, or R419H/R419H homotetramers. As clearly illustrated in Figure 4G,H and Table 2, a notable R419H-dependent modification effect was observed for both steady-state activation (G/G_max_) and inactivation (I/I_max_) properties of K_V_4.3 channels. For example, compared to the WT/WT homotetramer control, the steady-state activation (G/G_max_) curve of the WT/R419H heterotetramer and the R419H/R419H homotetramer was left-shifted by about 2.6 and 8.2 mV, respectively (Figure 4G), implying that increasing the relative proportion of the p.R419H variant in WT/R419H heterotetramers may lead to progressively higher K_V_4.3 channel open probability at the resting membrane potential. Similarly, the R419H-dependent right-shift of the steady-state K_V_4.3 channel inactivation (I/I_max_) curve (Figure 4H) suggests that increasing the relative proportion of the p.R419H variant may render WT/R419H heterotetramers less likely to be inactivated at the resting membrane potential. Together, these observations are consistent with the idea that, in WT/R419H heterotetramers, the p.R419H variant may exert a dominant effect on the voltage-dependent gating function of K_V_4.3 WT.

The superimposed region of steady-state activation and inactivation curves is known as the window current (Figure 4I–L), which correlates with the voltage range in which a significant fraction of inactivating ion channels may remain open with minimal inactivation, and therefore provides an estimate of the effective K_V_4.3 channel conductance under physiological conditions in neurons. In agreement with the foregoing R419H-dependent increase in K^+^ current amplitudes (Figure 4F), the WT/R419H heterotetramer and the R419H/R419H homotetramer displayed an enhancement of the size of the window current by about 2.8-fold and 3.6-fold, respectively (Figure 4J,L), indicating that the observed K^+^ current-potentiating effect can be in part attributed to the dominant gain-of-function voltage-dependent properties conferred by the p.R419H variant.

## 3. Discussion

Based on the standards and guidelines set forth by the American College of Medical Genetics (ACMG) [28], we employed multiple criteria to assess the clinical significance of the heterozygous *KCND3* c.1256G>A (p.R419H) variant identified in a patient with slowly progressive cerebellar ataxia, parkinsonism, cognitive dysfunction, and brain ion accumulation. Several lines of evidence support the pathogenicity of this *KCND3* variant: (i) this nonsynonymous variant in the *KCND3* gene, whose missense variants are frequently linked to cerebellar ataxia, contributes to a low allele frequency (<0.0001) in the population database gnomAD (Table 1) (criterion PP2 in the ACMG guideline); (ii) prediction of deleterious or damaging effects by multiple in silico bioinformatics analyses (Table 1) (criterion PP3); and (iii) the patient’s presentation of specific neurological symptoms relevant to *KCND3*-mutation-related SCA19/22 (criterion PP4). In addition, we provided the direct in vitro functional evidence (Figure 4) showing that the p.R419H variant is associated with a dominant gain-of-function effect on the K^+^ current amplitude and voltage-dependent gating of its K_V_4.3 WT counterpart, a strong indication of the variant’s pathogenicity (criterion PS3). Taken together, we propose that we have identified a “likely pathogenic” gain-of-function *KCND3* variant.

Table 3 outlines the genotype–phenotype relationship of known disease-associated *KCND3* variants. Patients with *KCND3*-related neurological disorders are characterized by heterogeneous clinical presentations including cerebellar ataxia, cognitive dysfunction, and movement disorders such as parkinsonism [10]. *KCND3*-related ataxia is further known to be associated with a wide range of disease onset (from very early ages to later stages of life), as well as distinctly different clinical courses (including episodic, non-progressive, and slowly progressive). Consistent with these notions, in the current study the patient harboring the heterozygous *KCND3* p.R419H variant presented with mild gait instability in his childhood and did not display complex neurological features until late adulthood. Moreover, the proband appears to be the only person in his family showing significant neurological disorders (Figure 1A), suggesting that the case is seemingly sporadic. Despite the fact that most *KCND3*-related disorders are autosomal dominant, de novo mutation and incomplete penentrance have been reported as well [6,9]. Therefore, the presence of four persons carrying the c.1256G>A (p.R419H) variant in gnomAD (Table 1) may additionally imply that this is a rare *KCND3* variant with incomplete penetrance.

To date, nearly 30 *KCND3* variants have been associated with neurological or cardiac disorders (Table 3). A majority of *KCND3* variants linked to neurological (e.g., SCA19/22) and cardiac (e.g., Brugada syndrome) disorders display loss-of-function and gain-of-function phenotypes, respectively, even though some of the cardiopathogenic gain-of-function *KCND3* mutations were later associated with neurological pathogenicty as well. The p.R419H variant identified in our ataxic patient exerts a dominant gain-of-function effect on functional K^+^ current level, which is probably attributed to a notable alteration of the steady-state voltage-dependent activation and inactivation of K_V_4.3 channels. Moreover, our ECG analysis reveals that the patient carrying the p.R419H variant does not appear to display detectable cardiac arrhythmia. Previously, another gain-of-function *KCND3* variant, p.L450F, was originally linked to the Brugada syndrome; however, this variant was later identified in a patient with cerebellar gait ataxia but no significant heart problems [11,14]. As far as cerebellar ataxia is concerned, the aforementioned observations support the idea that functional homeostasis of K_V_4.3 plays an imperative role in the operation of cerebellar physiology, and that both loss- and gain-of-function phenotypes of K_V_4.3 variant channels may considerably perturb neuronal excitability in the cerebellar circuit and therefore contribute to the pathogenesis of ataxia. Consistent with these notions, both loss- and gain-of-function variants in the *KCNC3* gene encoding another voltage-gated K^+^ channel (K_V_3.3) have been associated with spinocerebellar ataxia type 13 [47].

**Figure 5:**
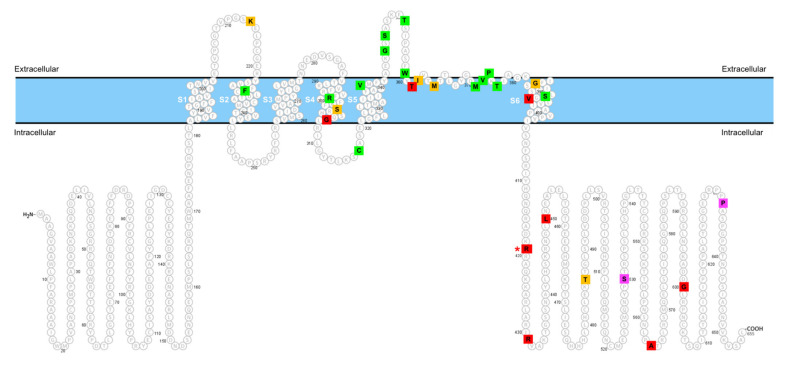
Topographic representation of disease-associated *KCND3* variants in the K_V_4.3 subunit. Green and red symbols represent K_V_4.3 variants with loss-of-function and gain-of-function phenotypes, respectively. Magenta symbols indicate K_V_4.3 variants with no significant alteration of functional properties. Yellow symbols refer to K_V_4.3 variants that have yet to be functionally characterized. The red asterisk denotes the p.R419H variant studied in the current report. See Table 3 for genotype–phenotype relationship.

Figure 5 summarizes the topographic localization of currently known disease-associated *KCND3* variants within the K_V_4.3 subunit. Interestingly, virtually all of the loss-of-function variants are located in the transmembrane region of the channel protein. In contrast, many of the gain-of-function variants, including the two ataxia-related gain-of-function variants p.R419H and p.L450F, are found in the cytoplasmic carboxyl-terminal region. It is unclear how the replacement of arginine with histidine at residue 419 may instigate such a significant gain-of-function effect on voltage-dependent gating of K_V_4.3 channel. The amino acid substitution at this evolutionary conserved K_V_4.3 residue is unlikely to dramatically affect the secondary protein structure of the proximal carboxyl-terminal region. Nonetheless, our in silico analyses suggest that p.R419 may be in close proximity with two intracellular domains, the S4-S5 linker and the amino-terminal region (Figure 2E), both essential for regulating voltage-dependent gating of K^+^ channels. Detailed experimental analyses will be required in the future to determine whether the histidine substitution may have a direct impact on the potential interaction between p.R419 and its structural microenvironment in K_V_4.3 channel.

As depicted in Figure 1B, brain MRI revealed significant iron deposition in bilateral caudate nuclei and lentiform nuclei of the basal ganglia, and in bilateral dentate nuclei of the cerebellum. It is an open question regarding the mechanistic link between K_V_4.3 current level and iron accumulation in neurons. Neuronal iron overload may result from enhanced postsynaptic iron uptake by the divalent metal transporter 1 (DMT1), and has been suggested to contribute to neurodegenerative diseases such as the Parkinson’s disease [48]. In addition, activation of K^+^ channels was shown to promote DMT1-mediated iron import into neuroblastoma cells [49]. We therefore speculate that the gain-of-function p.R419H variant may similarly potentiate DMT1-mediated iron uptake in specific regions in the brain. Since the iron-rich dentate nucleus serves as one of the largest deep cerebellar nuclei essential for the output signal from the cerebellum [50], dysregulation of iron homeostasis in bilateral dentate nuclei may lead to substantial anomaly of the cerebellar function. An analogous K_V_4.3 p.R419H-induced enhancement of iron uptake may also take place in bilateral lentiform and caudate nuclei of the basal ganglia, resulting in the parkinsonism observed in the patient. To the best of our knowledge, the current study provides the first evidence suggesting a potential association between K_V_4.3 gain-of-function and the susceptibility for brain iron accumulation. Taken as a whole, our findings highlight the wide variation in the phenotypical expression and pathophysiological outcome of disease-associated *KCND3* variants.

## 4. Materials and Methods

### 4.1. Patient Evaluations and Ethics

This study was supported by the Neurogenetics Translational Center of Excellence, Department of Neurology, UPenn. The patient was evaluated by two neurologists with expertise in movement disorders and neurogenetics at UPenn. Molecular tests, neurocognitive evaluation, and neuroimaging studies were conducted as part of clinical care. Informed consent was provided by the patient.

### 4.2. Genetic Analyses

Genomic/mitochondrial DNA was extracted from white blood cells in the peripheral venous blood. Screening of a neurodegeneration with brain iron accumulation gene panel was implemented by Associated Regional and University Pathologists (ARUP) laboratories (Salt Lake City, UT, USA). Whole exome sequencing (WES), mitochondrial sequencing, and deletion testing were conducted by the XomeDxPlus test of GeneDx (BioReference Laboratories, Gaithersburg, MD, USA) to detect disease-relevant variant. The targeted exonic regions and flanking splice junctions of the genome were simultaneously sequenced with 100 bp paired-end reads by massively parallel sequencing on an Illumina HiSeq 2000 sequencing system (NextGen Healthcare, Irvine, CA, USA). A customized analysis tool (Xome Analyzer, GeneDx; BioReference Laboratories, Gaithersburg, MD, USA) was utilized to assemble and align the bi-directional sequence to reference genome sequences (GRCh37/UCSC hg19), as well as calling for sequence variants in the regions of interest throughout the genome. The potentially pathogenic variants originally identified were further confirmed by an appropriate method such as capillary sequencing. Sequence alterations were reported based on the Human Genome Variation Society (HGVS) nomenclature guideline. The identified variants were subject to further evaluation by searching in gnomAD and the 1000 Genome Browser (1000 Genomes).

### 4.3. Bioinformatics Tools

CADD [23], PolyPhen-2 [24], SNPs&GO [25], SIFT [26], and MutationTaster [27] were employed to assess the pathogenicity of the *KCND3* variant. Amino acid sequences of K_V_4.3 homologs and orthologs from multiple species were aligned by using UniProt. UCSF Chimera [51] interfaced with Modeller [52] were applied to generate a K_V_4.3 homology model based on the crystal structure of the K_V_1.2 channel (PDB ID: 3LUT) [53].

### 4.4. cDNA Constructs

Amino-terminal Myc-tagged K_V_4.3 (Myc-K_V_4.3) was generated by subcloning human K_V_4.3 cDNA into the pcDNA3.1-Myc vector (Invitrogen, Carlsbad, CA, USA). The K_V_4.3 p.R419H variant was created by using the QuikChange Site-Directed Mutagenesis Kit (Stratagene, La Jolla, CA, USA). Amino-terminal HA-tagged KChIP2 and KChIP3 (HA-KChIP2 and HA-KChIP3) were created by subcloning human KChIP2 and KChIP3 cDNAs, respectively, into the pcDNA3-HA vector (Invitrogen, Carlsbad, CA, USA). All the constructs were verified by DNA sequencing. For in vitro transcription, appropriate restriction enzymes were applied to linearize cDNAs, from which capped cRNAs were transcribed using the Ambion mMessage mMachine T7 kit (Thermo Scientific, Waltham, MA, USA).

### 4.5. Cell Culture and Transfection

Human embryonic kidney (HEK) 293T cells were grown in Gibco Dulbecco’s modified Eagle’s medium (DMEM) (Thermo Scientific, Waltham, MA, USA) with 10% fetal bovine serum (Thermo Scientific, Waltham, MA, USA), 1 mM sodium pyruvate, 100 units/mL HyQ penicillin-streptomycin, and maintained at 37 °C in a humidified incubator with 95% air and 5% CO_2_. Cells were plated onto six- (for surface biotinylation) or 12-well plates (for total protein), or poly-D-lysine-coated coverslips in 24-well plates (for immunofluorescence) 24 h before transfection. Transient transfection was performed by the calcium phosphate method. Briefly, DNA/calcium phosphate precipitate was prepared by mixing one volume of DNA in 250 mM CaCl_2_ with an equal-volume 2X HEPES-buffered saline (HBS) [(in mM) 280 NaCl, 50 HEPES, 1.5 Na_2_HPO_4_, pH 7.0]. The calcium phosphate precipitate was allowed to form for 20 min in the dark at room temperature prior to being added to the cultures. The quantities of cDNA used for different experiments are as follows: 400 ng/well for immunofluorescence, 800 ng/well for total protein, and 1600 ng/well for surface biotinylation. The DNA/calcium phosphate precipitates were added drop-wise to cells, which were subject to 37 °C incubation for 3–4 h. To terminate the transfection, the mixture solution was replaced with fresh medium pre-warmed in the 37 °C incubator. The cells were returned to the 5% CO_2_ incubator at 37 °C until further processing. For co-expression experiments, cDNAs for individual K_V_4.3 and the auxiliary subunit were mixed in equimolar ratio.

### 4.6. Immunoblotting

Transfected cells were lysed in an ice-cold lysis buffer [(in mM) 150 NaCl, 5 EDTA, 50 Tris-HCl pH 7.6, 1% Triton X-100] containing a complete protease inhibitor cocktail (Roche Applied Science, Basel, Switzerland). After adding the Laemmli sample buffer to the lysates, samples were sonicated on ice (three times for five seconds each) and heated at 70 °C for 5 min for further process. Samples were then separated by 7.5% SDS-PAGE, electrophoretically transferred to nitrocellulose membranes, and detected using mouse anti-Myc (clone 9E10), or rabbit anti-α-tubulin (Bethyal Laboratories, Montgomery, TX, USA) antibodies. Blots were exposed to horseradish-peroxidase-conjugated goat anti-mouse IgG (Jackson ImmunoResearch, West Grove, PA, USA), or goat anti-rabbit IgG (Jackson ImmunoResearch, West Grove, PA, USA), and revealed by an enhanced chemiluminescence detection system (Thermo Scientific, Waltham, MA, USA). Acquisition of chemiluminescent signals from immunoblots was achieved by using the UVP AutoChemi image system (Ultra-Violet Products, Upland, CA, USA). Data shown are representative of at least 3 independent experiments. Densitometric scans of immunoblots were quantified with ImageJ (National Institute of Health, Bethesda, MD, USA).

For surface biotinylation analyses, transfected cells were incubated in 1 mg/mL sulfo-NHS-LC-biotin (Thermo Scientific, Waltham, MA, USA) in ice-cold phosphate-buffered saline (PBS) [(in mM) 136 NaCl, 2.5 KCl, 1.5 KH_2_PO_4_, 6.5 Na_2_HPO_4_, pH 7.4] with 0.9 mM CaCl_2_ and 0.5 mM MgCl_2_ at 4 °C for 1 h on orbital shaker. After the biotin reagents were removed, the cells were rinsed with glycine-containing PBS, followed by once in Tris-buffered saline [(in mM) 20 Tris-HCl, 150 NaCl, pH 7.4] to terminate biotinylation. Cells were solubilized in the lysis buffer. Cell lysates were incubated overnight at 4 °C with streptavidin-agarose beads (Thermo Scientific, Waltham, MA, USA). Beads were washed four times in the lysis buffer, followed by heating in Laemmli sample buffer to elute biotin-streptavidin complexes.

### 4.7. Immunofluorescence

HEK293T cells were seeded on poly-D-lysine-coated coverslips in 24-well culture dishes. Forty-eight hours after transfection, the coverslips containing HEK293T cells were fixed with 4% paraformaldehyde in PBS at room temperature for 20 min. Cells were permeabilized and blocked with a blocking buffer (5% normal goat serum in 20 mM phosphate buffer, pH 7.4, 0.1% (*v/v*) Triton X-100, and 450 mM NaCl) for 60 min at 4 °C. Appropriate dilutions of primary antibodies (1:200 for the mouse anti-Myc antibody; 1:200 for the rat anti-HA antibody) were appropriately applied in the blocking buffer overnight at 4 °C. Immunoreactivities were visualized with goat-anti-mouse antibodies conjugated to Alexa Fluor 488 (1:200; Invitrogen, Carlsbad, CA, USA), as well as goat-anti-rat antibodies conjugated to Alexa Fluor 633 (1:200; Invitrogen, Carlsbad, CA, USA) for 1 h at room temperature. Nuclei were labeled with DAPI. Finally, the coverslips were rinsed once in blocking buffer, twice in PBS, and twice in 0.1 M carbonate buffer, pH 9.2, before they were mounted on glass slides in a mounting medium (4% n-propyl gallate, 90% glycerol, 0.1 M carbonate, pH 9.2). A laser-scanning confocal microscope (Leica TCS SP8 STED; Wetzlar, Germany) was utilized to acquire fluorescence images.

### 4.8. Electrophysiological Analyses

*Xenopus* laevis oocytes (African Xenopus Facility, Knysna, South Africa) were used for functional studies. All animal procedures were in conformity with the animal protocol approved by the Institutional Animal Care and Use Committee of National Taiwan University. Frogs were anesthetized to dissect ovarian follicles, which were incubated in ND96 [(in mM): 96 NaCl, 2 KCl, 1.8 MgCl_2_, 1.8 CaCl_2_, and 5 HEPES, pH 7.2]. Stage V-VI oocytes were selected for cRNA injection, and the cRNAs for individual K_V_4.3 and KChIP2 were mixed in equimolar ratio. For analyzing K_V_4.3 WT/mutant heterotetramer, K_V_4.3 WT, mutant and KChIP2 were mixed in a molar ratio of 1:1:2. Injected oocytes were stored in ND96 at 16 °C for 2–3 days before being used for functional analyses. OC-725C oocyte clamp (Warner Instruments, Hamden, CT, USA) was used to record K^+^ currents through K_V_4.3 channels utilizing two-electrode voltage-clamp technology. The recording bath contained Ringer solution [(in mM): 3 KCl, 115 NaCl, 1.8 CaCl_2_, 10 HEPES, and 0.4 niflumic acid, pH 7.4 with methanesulfonic acid]. Borosilicate electrodes (0.1–1 MΩ) filled with 3 M KCl were used for voltage recording and current injection. Data were acquired and digitized via Digidata 1440A using pCLAMP 10.2 (Molecular Devices, San Jose, CA, USA). Oocytes were held at −90 mV and leak currents arising from passive membrane properties were subtracted by using the −P/4 method provided in the pCLAMP system. Data were obtained, normalized and analyzed as reported previously [54].

To generate peak K^+^ current–voltage curves for studying steady-state voltage-dependent activation of K_V_4.3, the voltage protocol comprised 500 ms test pulses stepped from −60 mV to +60 mV, in 10 mV increments. The relative conductance (G/G_max_) at a given test potential was calculated as G/G_max_ = ∆I_K_/∆I_K,+60_, where ∆I_K_ is current increment determined from peak current difference between adjacent test pulses, and ∆I_K,+60_ is the ∆I_K_ at the test potential +60 mV. A steady-state voltage-dependent activation curve was then generated by fitting the G/G_max_–voltage (G-V) curve with a Boltzmann equation: G/G_max_ = 1/{1 + exp[(*V*_0.5*a*_ *− V*)/*k_a_*]}, where *V_0_._5a_* is the half-activation voltage, and *k_a_* is the activation slope factor. To study the steady-state voltage-dependent inactivation of K_V_4.3, oocytes were subject to 1 s prepulses stepping from −120 to +10 mV with 10 mV increments, followed by a +60 mV test pulse for 500 ms. Normalized peak currents at +60 mV (I/I_max_) were then plotted against corresponding prepulse potentials. A steady-state voltage-dependent inactivation curve was then generated by fitting a I/I_max_ voltage curve with a Boltzmann function: I/I_max_ = 1/{1 + exp[(*V*_0.5*i*_ − *V*)/*k_i_*]}, where *V*_0.5*i*_ is the half-inactivation voltage, and *k_i_* is the inactivation slope factor. Window current analyses were determined from the apex and the area of the triangular overlap area between activation and inactivation curves.

### 4.9. Statistical Analyses

Statistical analyses were performed with Origin 7.0 (Microcal Software, Northampton, MA, USA). Numerical values were presented as mean ± SEM. The significance of the difference between two means was tested using Student’s *t*-test.

## Figures and Tables

**Table 1:** Bioinformatics analyses of the *KCND3* variant.

Variant at Chromosome Position: 1:112329579 C>T (GRCh37)		
Substitution: c.1256G>A; p.R419H (Reference Sequence: NM_004980.4)		
Tools	Results	Interpretation
^†^ gnomAD	Variant found in 4/251,408	Allele frequency: 1.591 × 10^−5^
^‡^ 1000 Genomes	Not identified	Absent in 1000 Genomes database
^§^ CADD	Phred score: 28.2	Top 0.15% most deleterious variant
^¶^ PolyPhen-2	Score: 1	Probably damaging
^††^ SNPs&GO	Reliability Index: 6	Disease-related
^‡‡^ SIFT	Score: 0.01	Deleterious (<0.05 deleterious)
^§§^ MutationTaster	Prob: 0.999999708078826	Disease causing

Remarks: ^†^ The *KCND3* c.1256G>A (p.R419H) variant was found in 4 out of 251,408 sequenced alleles (total population), equivalent to an estimated allele frequency of 1.591 × 10^−5^. For non-Finnish European population, the estimated allele frequency is 3.519 × 10^−5^. ^‡^ The *KCND3* variant was not found in 1000 genomes, in which a part of sequence data was not included in gnomAD. ^§^ The CADD Phred score is 28.2, suggesting that this *KCND3* variant is among the top 0.15% most deleterious variants of the human genome. ^¶^ The polyphen-2 score (ranging from 0 to 1) for the *KCND3* variant was calculated as 1.000, the highest score, suggestive of it probably being damaging. ^††^ The reliability index (ranging from 0 to 10) for the *KCND3* variant was 6, indicative of being disease-related with a high predicting confidence. ^‡‡^ The SIFT score for the *KCND3* variant was predicted to be less than 0.05, signifying it being notably damaging. ^§§^ This high probability score (close to 1) predicts that the *KCND3* variant is likely disease causing.

**Table 2:** Comparison of the K_V_4.3 voltage-dependent gating parameters of the wild type (WT) and the p.R419H variant. Steady-state activation and inactivation curves were generated from the averages of 9–12 cells expressing the indicated K_V_4.3 constructs. Data were subject to fitting with the Boltzmann equation as described in the Materials and Methods section and depicted in Figure 4. *V*_0_._5*a*_: half-activation voltage. *k_a_*: activation slope factor. *V_0_._5i_*: half-inactivation voltage. *k_i_*: inactivation slope factor.

(+KChIP2)	Activation		Inactivation	
	*V*_0.5*a*_ (mV)	*k_a_*	*V*_0.5*i*_ (mV)	*k_i_*
WT	−27.6	7.2	−56.3	6.1
WT/p.R419H	−30.2	7.6	−51.7	8.3
p.R419H	−35.8	5.7	−51.1	6.8

**Table 3:** The genotype–phenotype relationship for *KCND3* variants associated with neurological or cardiac disorders. Notation for in vitro functional phenotype: GOF, gain of function; LOF, loss of function; NSFC, no significant function change; n.a., not available. See Figure 5 for topographic representation.

*KCND3* Variant	Clinical Feature	Remark	In Vitro Function	Reference
p.K214R	Episodic gait disorder, vertigo, paraesthesia, pyramidal signs, abnormal ocular movement.	An asymptomatic mother carried the variant, suggesting incomplete penetrance.	n.a.	[29]
p.F227 deletion	Slowly progressive cerebellar ataxia, onset from teenage to middle age; oculomotor abnormalities, pyramidal signs parkinsonism, epilepsy, or cognitive impairment have been reported in some cases.	Recurrently identified in pedigrees with autosomal dominant inheritance from multiple ethnic groups.	LOF	[6,30,31]
p.R293_F295 duplication	Early onset cerebellar ataxia, intellectual disability, oral apraxia, and epilepsy.	De novo mutation.	LOF	[32]
p.S301P	Early onset forms with neurodevelopmental disorder, epilepsy, parkinsonism-dystonia, and ataxia in adulthood	Apparently de novo mutation.	n.a.	[10]
p.G306A	Cardiocerebral syndrome characterized by early repolarization syndrome in combination with refractory epilepsy, and intellectual disability.	De novo mutation.	GOF	[13]
p.C317Y	Cerebellar ataxia onset at teenage, developmental delay, intellectual disability, myoclonus, and dystonia.	De novo mutation.	LOF	[9]
p.V338E	Adult-onset cerebellar ataxia; cognitive dysfunction.	Identified from an autosomal dominant inheritance pedigree.	LOF	[6,9]
p.G345V	Adult-onset cerebellar ataxia; variable pyramidal signs and oculomotor abnormalities.	Identified in autosomal dominant pedigrees from multiple ethnic groups. Incomplete penetrance was reported in a pedigree.	LOF	[6,33]
p.S347W	Adult-onset slowly progressive cerebellar ataxia.	Undetermined inheritance.	LOF	[33]
p.T352P	Mild cerebellar ataxia, cognitive impairment; variable degree of oculomotor disturbance, neuropathy, tremor, and myoclonus.	Identified from a large pedigree with autosomal dominant cerebellar ataxia.	LOF	[7,34]
p.W359G	Congenital nonprogressive ataxia; hypotonia.	De novo mutation.	LOF	[33]
p.T361S	Early onset lone atrial fibrillation.	One single case identified from a cohort with atrial fibrillation.	GOF	[35]
p.I362M	Cerebellar ataxia.	Identified from a pedigree with autosomal dominant cerebellar ataxia.	n.a.	[36]
p.M365T	Cerebellar ataxia.	One single case identified in an autosomal dominant cerebellar ataxia cohort study.	n.a.	[36]
p.M373L	Adult-onset pure cerebellar ataxia.	Two affected individuals from an autosomal dominant inheritance pedigree.	LOF	[7]
p.V374A	Progressive cerebellar ataxia and bradyphrenia, cognitive impairment, paroxysmal ataxia exacerbations.	Two affected individuals from an autosomal dominant inheritance pedigree.	LOF	[37]
p.P375S	Teenage- or adult-onset cerebellar ataxia; cognitive dysfunction, dystonia, and bradykinesia.	A symptomatic mother–son pair from an autosomal dominant inheritance pedigree.	LOF	[9]
p.T377M	(i) Adolescence or adult-onset cerebellar ataxia; cognitive impairment in some patients. (ii) Hereditary spastic paraplegia.	A recurrently reported mutation identified in multiple ethnic groups. One single case identified from a cohort study of hereditary spastic paraplegia.	LOF	[6,9,38,39]
p.G384S	Cerebellar ataxia, intellectual disability, dystonia, and myoclonus.	De novo mutation.	n.a.	[40]
p.S390N	Teenage- or adult-onset cerebellar ataxia; cognitive dysfunction in some patients.	A recurrently reported mutation identified in multiple ethnic groups.	LOF	[7,41]
p.V392I	(i) Sudden unexplained death syndrome (ii) Dravet syndrome. (ii) Cerebellar ataxia, intellectual disability, epilepsy, early repolarization syndrome and paroxysmal atrial fibrillation.	Identified in a case with autopsy-negative sudden unexplained death syndrome at first; one single case with Dravet syndrome was linked to the variant; a pair of siblings presented with cardiocerebral syndrome.	GOF	[12,42,43]
p.R419H	Slowly progressive cerebellar ataxia, parkinsonism, and cognitive dysfunction.	Identified in an apparently sporadic case.	GOF	(current study)
p.R431C	Episodic ataxia.	One single case from a cohort study of episodic ataxia.	n.a.	[44,45]
p.R431H	Brugada syndrome.	Identified from a pedigree with Brugada syndrome.	GOF	[44]
p.L450F	(i) Brugada syndrome (ii) Late onset cerebellar ataxia, pyramidal signs.	Identified in cases with Brugada syndrome at first; one case with autosomal dominant cerebellar ataxia was later reported.	GOF	[11,14,36]
p.T486A	(i) Hereditary spastic paraplegia; (ii) Early-onset of persistent lone atrial fibrillation.	One single case identified from a cohort study of hereditary spastic paraplegia. Two individuals observed in the cohort study of early-onset of persistent lone atrial fibrillation.	n.a.	[39,46]
p.S530P	Autopsy-negative sudden unexplained death syndrome.	Identified from a cohort with sudden unexplained death syndrome.	NSFC	[42]
p.A564P	Early-onset of persistent lone atrial fibrillation.	Identified from a cohort study.	GOF.	[46]
p.G600R (p.G581R for the short isoform)	Brugada syndrome; autopsy-negative sudden unexplained death syndrome.	Recurrently observed from patients with Brugada syndrome or sudden unexplained death syndrome.	GOF	[11,42]
p.P633S (p.P614S for the short isoform)	Late onset cerebellar ataxia, decreased reflexes, and vibration sense.	One single case from a cohort study of cerebellar ataxia.	NSFC	[14,36]

## Data Availability

All data relevant to the study are included in the article or uploaded as Supplementary Information.

## Supplementary Materials

The following are available online at https://www.mdpi.com/article/10.3390/ijms22158247/s1, Supplementary Video S1. The clinical presentation of the patient harboring *KCND3* c.1256G>A (p.R419H) variant in this report. Neurological examination of the 69-year-old gentleman revealed cerebellar ataxia with mild parkinsonism, characterized by masked facial expression, perioral dyskinesias, mild intention tremor, bilateral dysrhythmokinesis, paratonia, lower greater than upper extremity bradykinesia, upright rigid posture, wide-based gait with short stride length, and mild tandem gait impairment. Supplementary Figure S1. Thumbnail image from Supplementary Video S1.

## Abbreviations

ACMG	American College of Medical Genetics
DMEM	Dulbecco’s modified Eagle’s medium
DMT1	divalent metal transporter 1
ECG	electrocardiogram
gnomAD	genome Aggregation Database
HBS	HEPES buffered saline
HEK	human embryonic kidney
K^+^	potassium
KChIP2/3	K^+^ channel interacting protein 2 and 3
PBS	phosphate-buffered saline
SCA19/22	spinocerebellar ataxia type 19 and 22
UPenn	University of Pennsylvania
WES	whole exome sequencing
WT	wild-type
