# Supplementary figures and images for: Rare Gain-of-Function KCND3 Variant Associated with Cerebellar Ataxia, Parkinsonism, Cognitive Dysfunction, and Brain Iron Accumulation
Source: Int J Mol Sci. 2021 Jul 31;22(15):8247. doi: 10.3390/ijms22158247 (PMC8347726; doi:10.3390/ijms22158247)

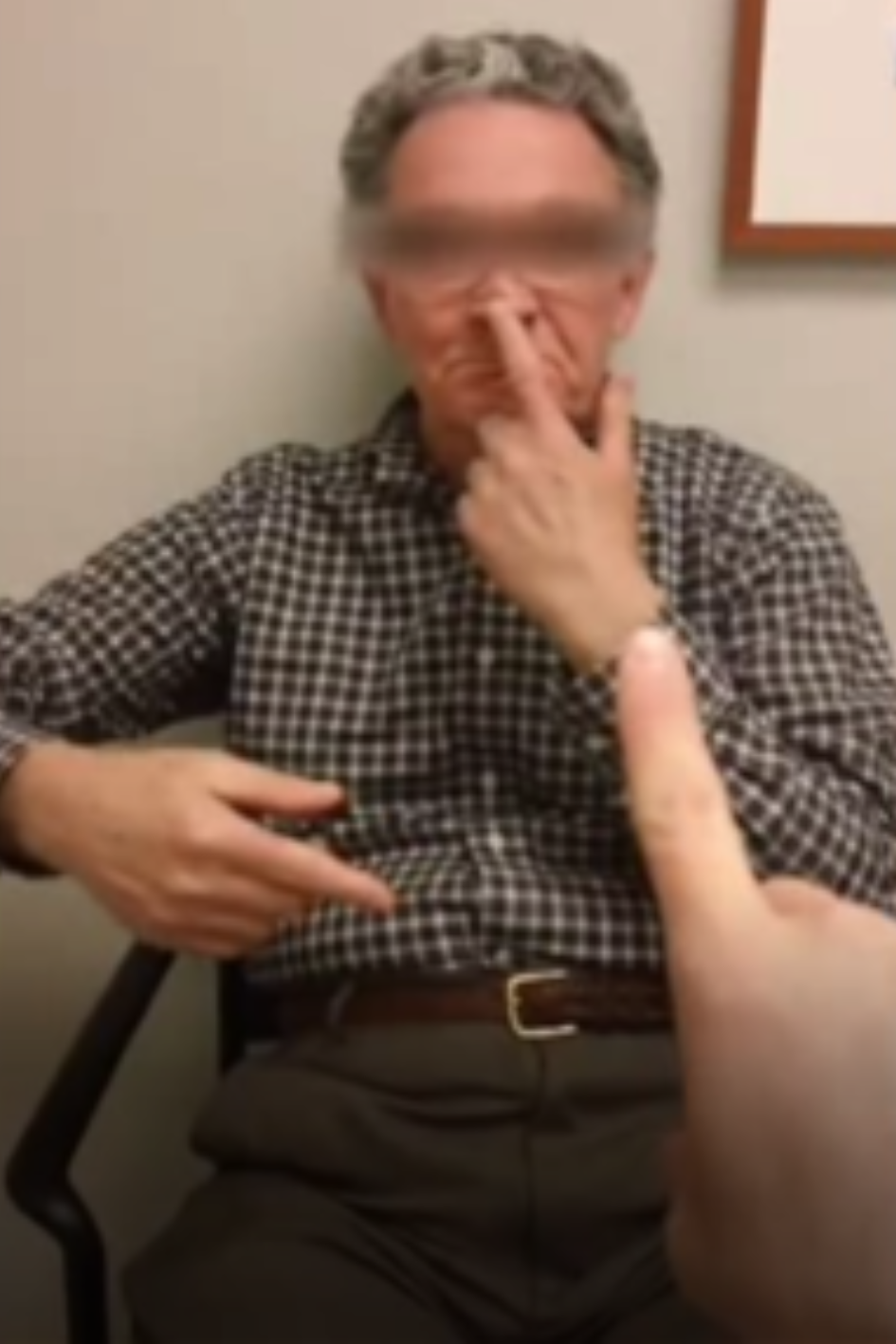

Supplement: Supplementary file 1 [file ijms-22-08247-s001.zip › Supplementary Figure S1.tif]
